# 5-Fluoro-3-(4-fluoro­phenyl­sulfin­yl)-2-methyl-1-benzofuran

**DOI:** 10.1107/S1600536810021823

**Published:** 2010-06-16

**Authors:** Hong Dae Choi, Pil Ja Seo, Byeng Wha Son, Uk Lee

**Affiliations:** a Department of Chemistry, Dongeui University, San 24 Kaya-dong Busanjin-gu, Busan 614-714, Republic of Korea; b Department of Chemistry, Pukyong National University, 599-1 Daeyeon 3-dong, Nam-gu, Busan 608-737, Republic of Korea

## Abstract

In the title compound, C_15_H_10_F_2_O_2_S, the S=O and the 4-fluoro­phenyl groups are located on opposite sides of the plane of benzofuran ring system, and the 4-fluoro­phenyl ring is nearly perpendicular to the benzofuran plane with a dihedral angle of 89.93 (4)°. In the crystal structure, mol­ecules are linked by weak inter­molecular C—H⋯O hydrogen bonding and C—H⋯π inter­actions.

## Related literature

For the pharmacological activity of benzofuran compounds, see: Aslam *et al.* (2006 ▶); Galal *et al.* (2009 ▶); Khan *et al.* (2005 ▶). For natural products with benzofuran rings, see: Akgul & Anil (2003 ▶); Soekamto *et al.* (2003 ▶). For the structures of related 3-(4-fluoro­phenyl­sulfin­yl)-2-methyl-1-benzofuran deriv­atives, see: Choi *et al.* (2010*a* ▶,*b* ▶).
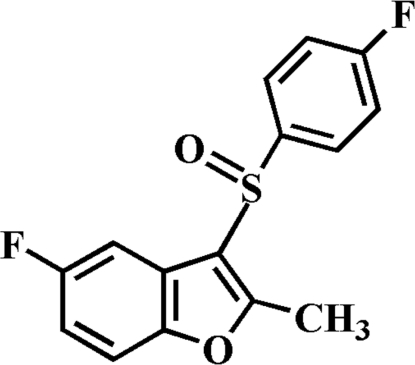


## Experimental

### 

#### Crystal data

C_15_H_10_F_2_O_2_S
*M* *_r_* = 292.29
Orthorhombic, 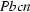

*a* = 14.9369 (4) Å
*b* = 10.6284 (3) Å
*c* = 16.1532 (4) Å
*V* = 2564.41 (12) Å^3^
*Z* = 8
Mo *K*α radiation
μ = 0.27 mm^−1^
*T* = 174 K
0.40 × 0.32 × 0.28 mm

#### Data collection

Bruker SMART APEXII CCD diffractometer
Absorption correction: multi-scan (*SADABS*; Bruker, 2009 ▶) *T*_min_ = 0.898, *T*_max_ = 0.927
22576 measured reflections
2962 independent reflections
2578 reflections with *I* > 2σ(*I*)
*R*_int_ = 0.029

#### Refinement

*R*[*F* ^2^ > 2σ(*F* ^2^)] = 0.031
*wR*(*F* ^2^) = 0.091
*S* = 1.08
2962 reflections
183 parameters
H-atom parameters constrained
Δρ_max_ = 0.29 e Å^−3^
Δρ_min_ = −0.32 e Å^−3^

### 

Data collection: *APEX2* (Bruker, 2009 ▶); cell refinement: *SAINT* (Bruker, 2009 ▶); data reduction: *SAINT*; program(s) used to solve structure: *SHELXS97* (Sheldrick, 2008 ▶); program(s) used to refine structure: *SHELXL97* (Sheldrick, 2008 ▶); molecular graphics: *ORTEP-3* (Farrugia, 1997 ▶) and *DIAMOND* (Brandenburg, 1998 ▶); software used to prepare material for publication: *SHELXL97*.

## Supplementary Material

Crystal structure: contains datablocks global, I. DOI: 10.1107/S1600536810021823/xu2777sup1.cif

Structure factors: contains datablocks I. DOI: 10.1107/S1600536810021823/xu2777Isup2.hkl

Additional supplementary materials:  crystallographic information; 3D view; checkCIF report

## Figures and Tables

**Table 1:** Hydrogen-bond geometry (Å, °) *Cg* is the centroid of the furan ring.

*D*—H⋯*A*	*D*—H	H⋯*A*	*D*⋯*A*	*D*—H⋯*A*
C9—H9*B*⋯O2^i^	0.98	2.45	3.200 (2)	133
C14—H14⋯O2^ii^	0.95	2.55	3.270 (2)	133
C15—H15⋯*Cg*^ii^	0.95	2.98	3.828 (2)	149

Symmetry codes: (i) 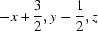; (ii) 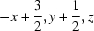.

**Fig. 1.**
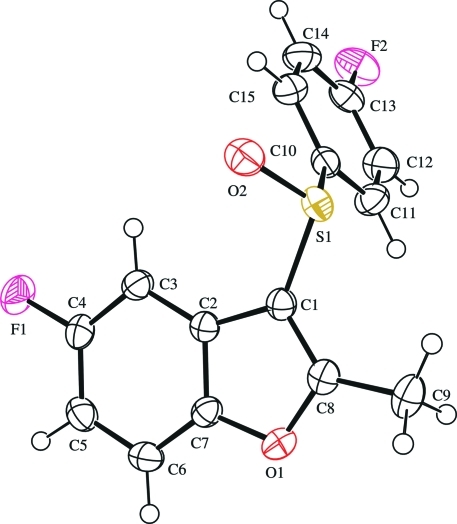
The molecular structure of the title compound with the atom numbering scheme. Displacement ellipsoids are drawn at the 50% probability level. H atoms are presented as a small spheres of arbitrary radius.

**Fig. 2.**
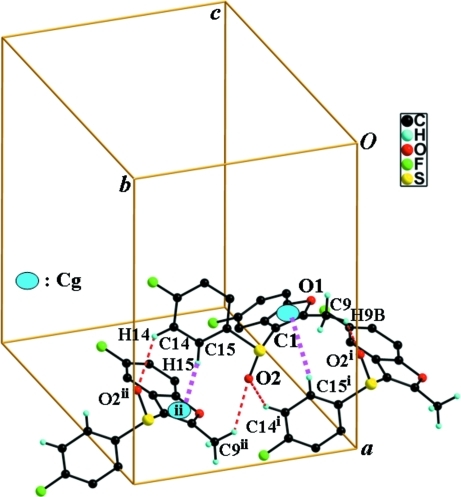
C–H···O and C–H···π interactions (dotted lines) in the crystal structure of the title compound. Cg denotes the ring centroid. [Symmetry codes: (i) - x + 3/2, y - 1/2, z; (ii) - x +3/2, y + 1/2, z.]

## supplementary crystallographic information

### Comment

The compounds containing benzofuran skeleton exhibit diverse pharmacological properties such as antifungal (Aslam *et al.*, 2006), antitumor and antiviral (Galal *et al.*, 2009), antimicrobial (Khan *et al.*, 2005) activities. These compounds widely occur in nature (Akgul & Anil, 2003; Soekamto *et al.*, 2003). As a part of our ongoing studies of the effect of side chain substituents on the solid state structures of 3-(4-fluorophenylsulfinyl)-2-methyl-1-benzofuran analogues (Choi *et al.*, 2010a,b), we report the crystal structure of the title compound (Fig. 1).

The benzofuran unit is essentially planar, with a mean deviation of 0.012 (1) Å from the least-squares plane defined by the nine constituent atoms. The 4-fluorophenyl ring is almost perpendicular to the plane of the benzofuran fragment [89.93 (4)°] and is tilted slightly towards it. The crystal packing (Fig. 2) is stabilized by weak intermolecular C–H···O hydrogen bonds; the first one between the methyl H atom and the oxygen of the S═O unit, with a C9–H9B···O2^i^, and the second one between the  4-fluorophenyl H atom and the oxygen of the S═O unit, with a C14–H14···O2^ii^, respectively (Table 1). The molecular packing (Fig. 2) is further stabilized by a C–H···π interaction between the 4-fluorophenyl H atom and the furan ring of an adjacent benzofuran system, with a C15–H15···Cg ^ii^(Table 1; Cg is the centroid of the C1/C2/C7/O1/C8 furan ring).

### Experimental

77% 3-Chloroperoxybenzoic acid (291 mg, 1.3 mmol) was added in small portions to a stirred solution of 5-fluoro-3-(4-fluorophenylsulfanyl)-2-methyl-1-benzofuran (350 mg, 1.2 mmol) in dichloromethane (30 ml) at 273 K. After being stirred at room temperature for 4 h, the mixture was washed with saturated sodium bicarbonate solution, the organic layer was separated, dried over magnesium sulfate, filtered and concentrated at reduced pressure. The residue was purified by column chromatography (hexane-ethyl acetate, 1:1 v/v) to afford the title compound as a colorless solid [yield 78%, m.p. 418-419 K; *R*_f_ = 0.49 (hexane-ethyl acetate, 1:1 v/v)]. Single crystals suitable for X-ray diffraction were prepared by slow evaporation of a solution of the title compound in benzene at room temperature.

### Refinement

All H atoms were positioned geometrically and refined using a riding model, with C–H = 0.93 Å for aryl and 0.96 Å for methyl H atoms. *U*_iso_(H) = 1.2*U*_eq_(C)  for aryl and 1.5*U*_eq_(C) for methyl H atoms.

### Crystal data

**Table:**

C_15_H_10_F_2_O_2_S	*F*(000) = 1200
*M**_r_* = 292.29	*D*_x_ = 1.514 Mg m^−^^3^
Orthorhombic, *P**b**c**n*	Mo *K*α radiation, λ = 0.71073 Å
Hall symbol: -P 2n 2ab	Cell parameters from 9962 reflections
*a* = 14.9369 (4) Å	θ = 2.4–27.6°
*b* = 10.6284 (3) Å	µ = 0.27 mm^−^^1^
*c* = 16.1532 (4) Å	*T* = 174 K
*V* = 2564.41 (12) Å^3^	Block, colourless
*Z* = 8	0.40 × 0.32 × 0.28 mm

### Data collection

**Table:**

Bruker SMART APEXII CCD diffractometer	2962 independent reflections
Radiation source: rotating anode	2578 reflections with *I* > 2σ(*I*)
graphite multilayer	*R*_int_ = 0.029
Detector resolution: 10.0 pixels mm^-1^	θ_max_ = 27.6°, θ_min_ = 2.4°
φ and ω scans	*h* = −19→19
Absorption correction: multi-scan (*SADABS*; Bruker, 2009)	*k* = −13→11
*T*_min_ = 0.898, *T*_max_ = 0.927	*l* = −18→21
22576 measured reflections	

### Refinement

**Table:**

Refinement on *F*^2^	Secondary atom site location: difference Fourier map
Least-squares matrix: full	Hydrogen site location: difference Fourier map
*R*[*F*^2^ > 2σ(*F*^2^)] = 0.031	H-atom parameters constrained
*wR*(*F*^2^) = 0.091	*w* = 1/[σ^2^(*F*_o_^2^) + (0.0448*P*)^2^ + 0.984*P*] where *P* = (*F*_o_^2^ + 2*F*_c_^2^)/3
*S* = 1.08	(Δ/σ)_max_ < 0.001
2962 reflections	Δρ_max_ = 0.29 e Å^−^^3^
183 parameters	Δρ_min_ = −0.32 e Å^−^^3^
0 restraints	Extinction correction: *SHELXL97* (Sheldrick, 2008), Fc^*^=kFc[1+0.001xFc^2^λ^3^/sin(2θ)]^-1/4^
Primary atom site location: structure-invariant direct methods	Extinction coefficient: 0.0035 (5)

### Special details

**Table:**

Geometry. All esds (except the esd in the dihedral angle between two l.s. planes) are estimated using the full covariance matrix. The cell esds are taken into account individually in the estimation of esds in distances, angles and torsion angles; correlations between esds in cell parameters are only used when they are defined by crystal symmetry. An approximate (isotropic) treatment of cell esds is used for estimating esds involving l.s. planes.
Refinement. Refinement of F^2^ against ALL reflections. The weighted R-factor wR and goodness of fit S are based on F^2^, conventional R-factors R are based on F, with F set to zero for negative F^2^. The threshold expression of F^2^ > 2sigma(F^2^) is used only for calculating R-factors(gt) etc. and is not relevant to the choice of reflections for refinement. R-factors based on F^2^ are statistically about twice as large as those based on F, and R- factors based on ALL data will be even larger.

### Fractional atomic coordinates and isotropic or equivalent isotropic displacement parameters (Å^2^)

**Table:**

	*x*	*y*	*z*	*U*_iso_*/*U*_eq_	
S1	0.66618 (2)	0.39412 (3)	0.07071 (2)	0.02868 (12)	
F1	0.74089 (8)	0.39246 (10)	0.42659 (5)	0.0468 (3)	
F2	0.43716 (7)	0.81769 (9)	0.16672 (7)	0.0533 (3)	
O1	0.59409 (7)	0.09156 (9)	0.19275 (6)	0.0308 (2)	
O2	0.76049 (7)	0.43398 (11)	0.08603 (7)	0.0371 (3)	
C1	0.63924 (9)	0.27918 (12)	0.14445 (8)	0.0257 (3)	
C2	0.65824 (8)	0.27675 (12)	0.23194 (8)	0.0244 (3)	
C3	0.69806 (9)	0.35842 (13)	0.28866 (9)	0.0287 (3)	
H3	0.7198	0.4390	0.2730	0.034*	
C4	0.70389 (10)	0.31507 (14)	0.36869 (9)	0.0317 (3)	
C5	0.67501 (10)	0.19769 (15)	0.39509 (9)	0.0334 (3)	
H5	0.6817	0.1735	0.4514	0.040*	
C6	0.63635 (10)	0.11614 (13)	0.33859 (9)	0.0318 (3)	
H6	0.6159	0.0350	0.3544	0.038*	
C7	0.62909 (8)	0.15881 (12)	0.25823 (8)	0.0265 (3)	
C8	0.60257 (9)	0.16619 (13)	0.12435 (9)	0.0287 (3)	
C9	0.57225 (10)	0.11157 (15)	0.04501 (10)	0.0379 (4)	
H9A	0.5068	0.1058	0.0448	0.057*	
H9B	0.5979	0.0273	0.0384	0.057*	
H9C	0.5919	0.1654	−0.0008	0.057*	
C10	0.59618 (9)	0.51953 (13)	0.10727 (8)	0.0275 (3)	
C11	0.50426 (10)	0.50221 (15)	0.11532 (10)	0.0354 (3)	
H11	0.4786	0.4215	0.1066	0.043*	
C12	0.45059 (10)	0.60330 (15)	0.13609 (11)	0.0401 (4)	
H12	0.3877	0.5934	0.1423	0.048*	
C13	0.49058 (11)	0.71859 (14)	0.14756 (10)	0.0364 (3)	
C14	0.58090 (11)	0.73846 (14)	0.13999 (10)	0.0391 (4)	
H14	0.6061	0.8195	0.1488	0.047*	
C15	0.63445 (10)	0.63648 (14)	0.11909 (9)	0.0342 (3)	
H15	0.6972	0.6471	0.1129	0.041*	

### Atomic displacement parameters (Å^2^)

**Table:**

	*U*^11^	*U*^22^	*U*^33^	*U*^12^	*U*^13^	*U*^23^
S1	0.03005 (19)	0.0329 (2)	0.02308 (18)	0.00313 (13)	−0.00015 (12)	0.00211 (12)
F1	0.0588 (6)	0.0527 (6)	0.0289 (5)	−0.0174 (5)	−0.0098 (4)	−0.0059 (4)
F2	0.0614 (6)	0.0369 (5)	0.0617 (7)	0.0204 (5)	0.0077 (5)	0.0049 (5)
O1	0.0328 (5)	0.0259 (5)	0.0338 (5)	−0.0047 (4)	−0.0004 (4)	−0.0046 (4)
O2	0.0271 (5)	0.0422 (6)	0.0421 (6)	0.0011 (4)	0.0044 (4)	0.0098 (5)
C1	0.0250 (6)	0.0265 (6)	0.0257 (6)	0.0018 (5)	−0.0009 (5)	−0.0013 (5)
C2	0.0227 (6)	0.0250 (6)	0.0256 (6)	0.0021 (5)	0.0002 (5)	−0.0009 (5)
C3	0.0305 (7)	0.0261 (6)	0.0294 (7)	−0.0034 (5)	−0.0007 (5)	−0.0014 (5)
C4	0.0312 (7)	0.0363 (7)	0.0275 (7)	−0.0027 (6)	−0.0048 (5)	−0.0038 (6)
C5	0.0342 (7)	0.0388 (8)	0.0273 (7)	0.0008 (6)	−0.0016 (6)	0.0063 (6)
C6	0.0331 (7)	0.0272 (7)	0.0352 (8)	−0.0009 (5)	0.0024 (6)	0.0053 (6)
C7	0.0248 (6)	0.0244 (6)	0.0302 (7)	0.0002 (5)	−0.0003 (5)	−0.0031 (5)
C8	0.0256 (6)	0.0302 (7)	0.0302 (7)	0.0024 (5)	−0.0005 (5)	−0.0039 (5)
C9	0.0349 (8)	0.0414 (8)	0.0373 (8)	−0.0003 (6)	−0.0064 (6)	−0.0134 (6)
C10	0.0292 (6)	0.0289 (7)	0.0244 (6)	0.0016 (5)	−0.0027 (5)	0.0035 (5)
C11	0.0285 (6)	0.0306 (7)	0.0471 (9)	−0.0018 (6)	−0.0034 (6)	0.0011 (6)
C12	0.0295 (7)	0.0414 (9)	0.0493 (10)	0.0042 (6)	−0.0001 (7)	0.0055 (7)
C13	0.0435 (8)	0.0303 (7)	0.0352 (8)	0.0101 (6)	0.0008 (6)	0.0064 (6)
C14	0.0477 (9)	0.0267 (7)	0.0429 (9)	−0.0032 (6)	0.0018 (7)	0.0028 (6)
C15	0.0318 (7)	0.0334 (7)	0.0374 (8)	−0.0051 (6)	0.0005 (6)	0.0041 (6)

### Geometric parameters (Å, °)

**Table:**

S1—O2	1.492 (1)	C6—C7	1.379 (2)
S1—C1	1.753 (1)	C6—H6	0.9500
S1—C10	1.794 (1)	C8—C9	1.478 (2)
F1—C4	1.363 (2)	C9—H9A	0.9800
F2—C13	1.357 (2)	C9—H9B	0.9800
O1—C8	1.366 (2)	C9—H9C	0.9800
O1—C7	1.379 (2)	C10—C15	1.381 (2)
C1—C8	1.359 (2)	C10—C11	1.391 (2)
C1—C2	1.442 (2)	C11—C12	1.382 (2)
C2—C7	1.393 (2)	C11—H11	0.9500
C2—C3	1.395 (2)	C12—C13	1.376 (2)
C3—C4	1.375 (2)	C12—H12	0.9500
C3—H3	0.9500	C13—C14	1.371 (2)
C4—C5	1.387 (2)	C14—C15	1.389 (2)
C5—C6	1.385 (2)	C14—H14	0.9500
C5—H5	0.9500	C15—H15	0.9500
			
O2—S1—C1	107.57 (6)	C1—C8—C9	132.66 (14)
O2—S1—C10	106.55 (7)	O1—C8—C9	116.41 (13)
C1—S1—C10	99.22 (6)	C8—C9—H9A	109.5
C8—O1—C7	106.51 (10)	C8—C9—H9B	109.5
C8—C1—C2	107.32 (12)	H9A—C9—H9B	109.5
C8—C1—S1	123.09 (10)	C8—C9—H9C	109.5
C2—C1—S1	129.30 (10)	H9A—C9—H9C	109.5
C7—C2—C3	119.53 (12)	H9B—C9—H9C	109.5
C7—C2—C1	104.67 (11)	C15—C10—C11	120.97 (13)
C3—C2—C1	135.77 (13)	C15—C10—S1	118.22 (11)
C4—C3—C2	115.84 (13)	C11—C10—S1	120.50 (11)
C4—C3—H3	122.1	C12—C11—C10	119.48 (14)
C2—C3—H3	122.1	C12—C11—H11	120.3
F1—C4—C3	117.95 (13)	C10—C11—H11	120.3
F1—C4—C5	117.26 (13)	C13—C12—C11	118.25 (14)
C3—C4—C5	124.79 (13)	C13—C12—H12	120.9
C6—C5—C4	119.34 (13)	C11—C12—H12	120.9
C6—C5—H5	120.3	F2—C13—C14	118.64 (14)
C4—C5—H5	120.3	F2—C13—C12	117.82 (14)
C7—C6—C5	116.56 (13)	C14—C13—C12	123.53 (14)
C7—C6—H6	121.7	C13—C14—C15	117.92 (14)
C5—C6—H6	121.7	C13—C14—H14	121.0
C6—C7—O1	125.52 (12)	C15—C14—H14	121.0
C6—C7—C2	123.92 (13)	C10—C15—C14	119.84 (14)
O1—C7—C2	110.55 (12)	C10—C15—H15	120.1
C1—C8—O1	110.93 (12)	C14—C15—H15	120.1
			
O2—S1—C1—C8	−129.33 (12)	C1—C2—C7—O1	−0.14 (14)
C10—S1—C1—C8	119.93 (12)	C2—C1—C8—O1	1.61 (15)
O2—S1—C1—C2	43.56 (14)	S1—C1—C8—O1	175.85 (9)
C10—S1—C1—C2	−67.18 (13)	C2—C1—C8—C9	−177.80 (14)
C8—C1—C2—C7	−0.87 (14)	S1—C1—C8—C9	−3.6 (2)
S1—C1—C2—C7	−174.64 (10)	C7—O1—C8—C1	−1.69 (15)
C8—C1—C2—C3	176.94 (15)	C7—O1—C8—C9	177.83 (12)
S1—C1—C2—C3	3.2 (2)	O2—S1—C10—C15	17.87 (13)
C7—C2—C3—C4	−1.17 (19)	C1—S1—C10—C15	129.42 (12)
C1—C2—C3—C4	−178.74 (14)	O2—S1—C10—C11	−168.52 (11)
C2—C3—C4—F1	−178.89 (12)	C1—S1—C10—C11	−56.97 (13)
C2—C3—C4—C5	1.3 (2)	C15—C10—C11—C12	−0.5 (2)
F1—C4—C5—C6	179.52 (13)	S1—C10—C11—C12	−173.95 (12)
C3—C4—C5—C6	−0.7 (2)	C10—C11—C12—C13	0.4 (2)
C4—C5—C6—C7	−0.1 (2)	C11—C12—C13—F2	179.03 (14)
C5—C6—C7—O1	178.89 (12)	C11—C12—C13—C14	−0.4 (3)
C5—C6—C7—C2	0.2 (2)	F2—C13—C14—C15	−179.02 (14)
C8—O1—C7—C6	−177.78 (13)	C12—C13—C14—C15	0.4 (2)
C8—O1—C7—C2	1.10 (14)	C11—C10—C15—C14	0.5 (2)
C3—C2—C7—C6	0.5 (2)	S1—C10—C15—C14	174.11 (12)
C1—C2—C7—C6	178.75 (13)	C13—C14—C15—C10	−0.5 (2)
C3—C2—C7—O1	−178.39 (11)		

### Hydrogen-bond geometry (Å, °)

**Table:**

Cg is the centroid of the furan ring.

**Table:**

*D*—H···*A*	*D*—H	H···*A*	*D*···*A*	*D*—H···*A*
C9—H9B···O2^i^	0.98	2.45	3.200 (2)	133
C14—H14···O2^ii^	0.95	2.55	3.270 (2)	133
C15—H15···Cg^ii^	0.95	2.98	3.828 (2)	149

Symmetry codes: (i) −*x*+3/2, *y*−1/2, *z*; (ii) −*x*+3/2, *y*+1/2, *z*.
